# Effects and mechanisms of exercise timing on cognitive function in older type 2 diabetes mellitus patients with mild cognitive impairment: A magnetic resonance imaging-based randomized controlled trial protocol

**DOI:** 10.1371/journal.pone.0361868

**Published:** 2026-11-04

**Authors:** Yanyu Lin, Zhiying Ma, Jinghua Zhang, Jie Zhuang

**Affiliations:** 1 School of Exercise and Health, Shanghai University of Sport, Shanghai, China; 2 College of Physical Education and Health, Shanghai Business School, Shanghai, China; Kyung Hee University, KOREA, REPUBLIC OF

## Abstract

**Background:**

Mild cognitive impairment (MCI) is a prevalent complication of type 2 diabetes mellitus (T2DM). Cognitive impairment progressively impairs self-care abilities, thereby triggering a vicious cycle of poor glycemic control and further cognitive decline. Exercise has been widely recognized as an effective strategy to improve both glycemic control and cognitive function; however, an optimal time of day for exercise to mitigate this neurodegenerative trajectory has not been determined. This trial aims to determine the most effective exercise timing to maximize cognitive benefits in older T2DM patients with MCI (T2DM-MCI) and to investigate the underlying neurobiological mechanisms using multimodal magnetic resonance imaging.

**Methods:**

In this randomized controlled trial, patients 60–75 years of age with T2DM‐MCI will be assigned to a morning exercise group (09:00–10:10), an evening exercise group (19:00–20:10), or a health education group. The exercise groups will complete a 12-week, home-based, remotely supervised exercise program comprising aerobic and resistance exercises. The health education group will receive a 12-week health education program. Cognitive function will be evaluated at baseline and post-intervention using the Montreal Cognitive Assessment, Color-Word Stroop Test, N-back task, Trail making Test, Attention Network Test, and Digit Symbol Substitution Test. Additionally, brain structure and function based on imaging, blood biomarkers, and physical fitness will also be assessed.

**Discussion:**

In this study, we aim to identify the optimal time of day for exercise to maximize cognitive benefits in patients with T2DM‐MCI and to elucidate underlying neurocognitive mechanisms. We will examine whether glycemic control mediates the relationship between exercise and cognitive function improvement. This research is expected to provide “time-specific” evidence for exercise prescription to maximize the neurocognitive benefits and mitigate dementia risk in this population.

**Trial registration:**

Chinese Clinical Trial Registry: [http://www.chictr.org.cn], identifier [ChiCTR2300073737].

## 1. Introduction

Cognitive dysfunction is increasingly recognized as an age-dependent complication of type 2 diabetes mellitus (T2DM) [1,2]. The prevalence of mild cognitive impairment (MCI) is estimated to be 45.0% among individuals with T2DM [3]. T2DM-associated MCI profoundly impairs patients’ self-management abilities, potentially accelerating disease progression [4,5]. MCI represents a modifiable stage between normal cognitive aging and dementia [6], and good glycemic control may help reverse impairments [7]. Thus, this pre-dementia stage serves as a critical window for therapeutic intervention to improve cognitive function in patients with T2DM [8].

Human cognitive function is modulated by the circadian rhythm system, exhibiting specific diurnal variation [9–12]. Cognitive performance varies by the time of day [13,14]. Furthermore, circadian rhythms disruption, characterized by abnormal diurnal patterns and altered clock gene expression, has been observed in individuals with T2DM and Alzheimer’s disease [15,16]. The loss of circadian homeostasis exerts detrimental effects on overall health, including metabolic disturbances [17], accelerated cognitive decline, and brain atrophy [18]. A bidirectional relationship exists between circadian disruption and neurodegeneration, suggesting that circadian rhythms may play an important role in the progression of neurodegenerative diseases [15,18,19].

Exercise has been shown to improve cognitive function and reduce the severity of cognitive impairment in older T2DM patients with MCI (T2DM-MCI). Improved cognition may enhance self-management capability and treatment adherence, ultimately decelerating the progression of T2DM-related complications [20–23]. In addition, exercise serves as an important external cue for regulating the circadian rhythm system and act as a circadian synchronizer [24]. By improving circadian regulation, exercise promotes healthy aging and mitigate age-related physiological decline [25–27]. Researchers have proposed that the time of day for exercise should be aligned with circadian rhythms to maximize health benefits, implying the presence of an optimal exercise timing window [26,28]. Growing evidence indicates that the physiological benefits of exercise may depend on the time of day at which it is performed. A meta‑analysis involving adults across varying health conditions have shown that afternoon or evening exercise produces greater improvements in glucose control than morning exercise [29]. Importantly, exercise may improve cognitive function, at least in part, through glycemic modulation [30–32]. Therefore, identifying the optimal time of day for exercise to improve glycemic control is clinically relevant for older adults with T2DM-MCI. However, it remains unclear whether a specific time of day is most beneficial for exercise-induced cognitive improvement. Clarifying the optimal exercise timing for cognitive benefits is equally important and warrants rigorous investigation.

Some studies have investigated the optimal time of day for exercise to improve cognitive function. Ingham-Hill et al. [33] and Khemila et al. [34] found that acute high-intensity exercise may be more effective in improving cognitive function in the morning than in the afternoon among adolescents. However, the results of Hasni et al. [35] and Maeneja et al. [36] did not support a significant time of day effect on the attention-related cognitive benefits of exercise. Overall, existing evidence on the optimal time of day for to confer cognitive benefits remains limited, with highly heterogeneous findings attributable to variations in study populations and exercise protocols. Additionally, there is a lack of studies examining long‑term cognitive adaptations to exercise performed at different times of day in adults with T2DM-MCI. Further research is needed to identify the optimal time of day for exercise and to explore the potential neural mechanisms using magnetic resonance imaging (MRI).

Therefore, the primary objective of this study is to compare the effectiveness of morning versus evening exercise for improving cognitive function in older T2DM‐MCI patients. The secondary objectives are: (1) to determine whether morning exercise and evening exercise induce differential changes in brain structure and function, including brain volume, functional connectivity, and white matter integrity; and (2) to examine whether improved glycemic control mediate the effects of exercise intervention on cognitive function, brain structure and function.

## 2. Materials and methods

### 2.1 Overview of study design

This is a three-arm, parallel-group, randomized controlled study that will be conducted at Shanghai University of Sport (Shanghai, China). Participants will be randomly assigned to one of three groups: morning exercise (ME), evening exercise (EE) or health education (HE). Participants in the exercise groups will receive a 12-week supervised training program combining aerobic and resistance exercise either from 09:00–10:10 (ME group) or 19:00–20:10 (EE group). The HE group will attend a health education course. All participants will be instructed to maintain their usual daily routine throughout the study period. Outcome assessments will be conducted at three timepoints: pre-intervention (week 0) and post-intervention (week 12). Fig 1 shows a detailed schedule of the participant enrolment, intervention, and assessments. And Fig 2 shows the study flowchart.

**Fig 1:**
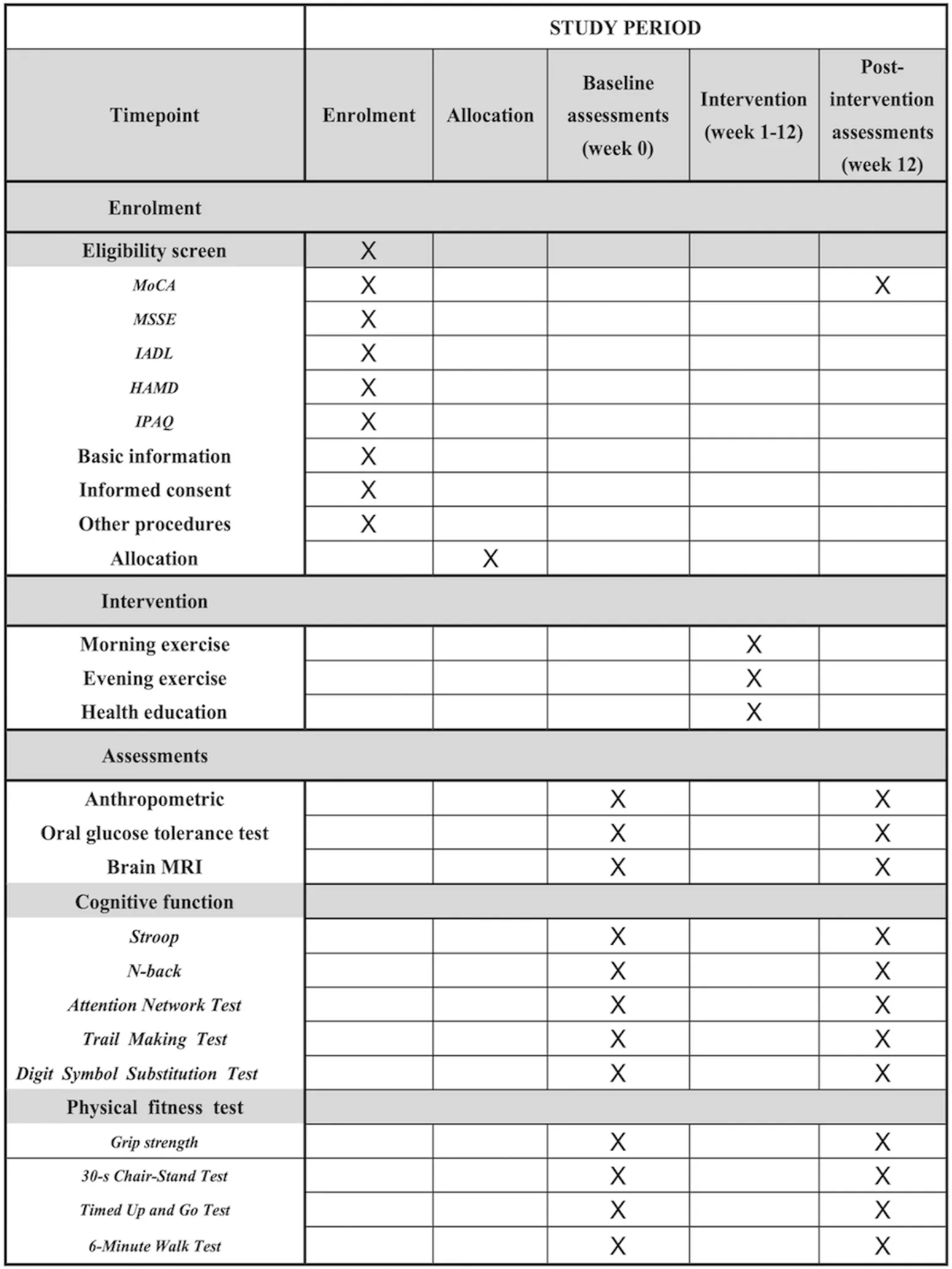
Participants timeline schematic. Abbreviations: MoCA, Montreal Cognitive Assessment; MMSE, Mini-Mental State Examination; IADL, Instrumental Activities of Daily Living; HAMD, Hamilton Depression Rating Scale; IPAQ, International Physical Activity Questionnaire; MRI: magnetic resonance imaging.

**Fig 2:**
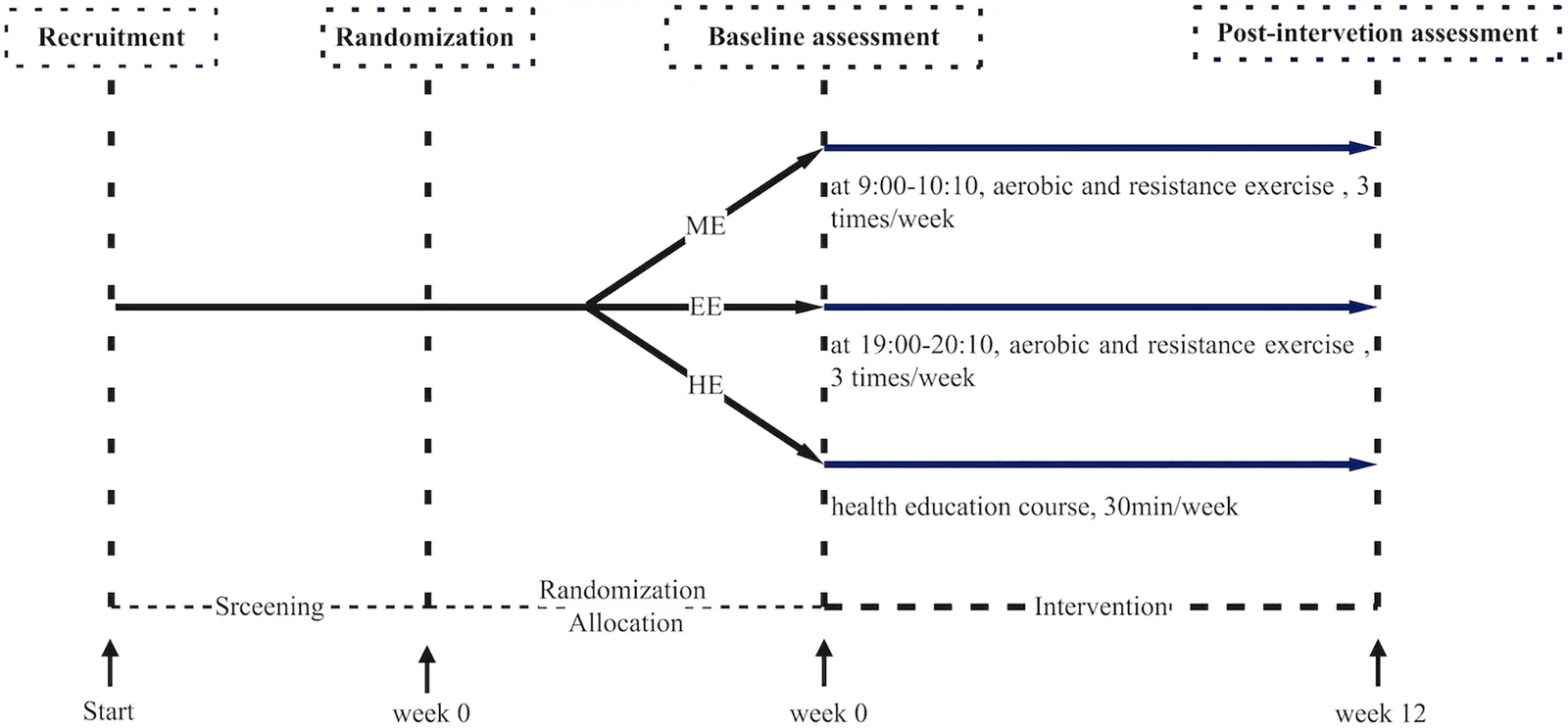
Flowchart of participants during the trial. Abbreviations: ME: morning exercise; EE: evening exercise; HE: health education.

#### Study hypotheses.

Hypothesis 1: Evening exercise will yield greater improvements in cognitive function, brain function, brain structure, and glycemic control than morning exercise.

Hypothesis 2: Glycemic control will mediate the beneficial effects of exercise on cognitive function and brain health.

### 2.2 Participants

The participants will be recruited from local hospitals and communities in Shanghai, China, via posters, health lectures, and word-of-mouth communication among researchers. Recruitment taking place from August 15, 2024, to October 30, 2026. Physicians will screen potential participants using the eligibility criteria, provide a brief introduction to the study, and refer eligible participants to the research team. The researchers will then provide detailed information about the study, confirm each individual’s willingness to participate, and obtain written informed consent. Our study was approved by the ethics committee of Shanghai University of Sport (number: 102772022RT078, date: 15 May 2023) and registered in the Chinese Clinical Trial Registry (number: ChiCTR2300073737, date: 19 July 2023). The study will be conducted in accordance with the Declaration of Helsinki. As of August 18, 2026, a total of 56 participants had been enrolled.

The eligibility criteria are as follows: (1) 60–75 years of age; (2) clinical diagnosis of T2DM and meeting Peterson’s criteria for MCI [37], including a subjective memory complaint, Montreal Cognitive Assessment (MoCA) score <26 and Mini-Mental State Examination score ≥  24; (3) fully capable of self-care, with an instrumental activities of daily living score ≥15; (4) no severe complications of T2DM; and (5) physical inactivity during the past 3 months (International Physical Activity Questionnaire: moderate-to-vigorous intensity physical activity < 150 min/week of moderate intensity or < 75 min/week of vigorous intensity physical activity).

The exclusion criteria are as follows: (1) neurodegenerative diseases (e.g., dementia, Parkinson’s disease, cerebrovascular disease); (2) other physical conditions that may interfere with the intervention and assessments, such as severe heart disease, liver disease, or kidney disease; (3) have a history of recurrent hypoglycemia; (4) mental illness or a Hamilton Depression scale score > 7; (5) alcohol abuse; (6) dyskinesia; and (7) fewer than 6 years of education.

Participants with contraindications to MRI will not be excluded from the overall study; however, MRI scanning will not be performed in these individuals.

Participation will be discontinued if any of the following occur: (1) voluntary withdrawal; (2) severe adverse events during the exercise intervention period, such as fractures and major cardiovascular events; or (3) the development of incidental clinical conditions, such as cancer and stroke, that may interfere with the assessment of outcomes.

### 2.3 Randomization and masking

Following baseline assessments, the eligible participants will be randomly allocated to ME, EE, and HE groups in a 1:1:1 ratio using block randomization with a block size of six. The randomization sequence will be generated by an independent statistician and implemented through an independent online computerized randomization system (https://www.sealedenvelope.com/simple-randomiser/v1/lists). Owing to the nature of the exercise intervention, masking of participants and fitness coaches is not feasible; however, outcome assessors and data analysis will remain blinded to group allocation. In addition, fitness coaches involved in the intervention will be separated from the outcome assessors to ensure the integrity of the blinding process. The study will be unblinded only after the completion of the statistical analysis.

### 2.4 Intervention

Participants in the ME and EE groups will complete home-based exercise through the Tencent Meeting platform, which will be used to stream the exercise intervention videos in real time. All exercise sessions will be performed in the post‑prandial state. Considering that the participants are physically inactive older adults, the entire exercise intervention will be divided into two phases: Stage 1, an exercise‑adaptation phase, and Stage 2, a maintenance phase. As shown in Table 1, the 12-week intervention will comprise three non-consecutive sessions per week. Each session will comprise 5 minutes of warm-up, 30 minutes of aerobic exercise, 30 minutes of resistance exercise, and 5 minutes of cool-down.

**Table 1:** Multicomponent exercise protocol.

Stage	Multicomponent exercise protocol	
Stage 1 1-4week	Frequency: 3 sessions per week	Warm-up: 5 min AE:40%−50% HRR for 30 min RE:2 set, 15RM, 10 Movements Cool-down: 5 min
Stage 2 5-12week	Frequency: 3 sessions per week	Warm-up: 5 min AE:50%−60% HRR for 30 min RE:2 set, 12RM, 10 Movements Cool-down: 5 min

Abbreviations: AE, aerobic exercise; RE, resistance exercise; RM, repetition Maximum; HRR, Heart rate reserve.

The aerobic exercise will consist of music-guided calisthenics performed at 40–60% heart rate reserve (HRR). Aerobic exercise intensity will be set at 40–50% heart rate reserve (HRR) in Stage 1, and increased to 50–60% HRR in Stage 2. This protocol has been from Watanabe et al. [38], with video playback speed adjusted monthly to maintain the target intensity. The resistance exercise will involve full-body training using elastic bands. Each resistance exercise session will be performed for two sets of 15 repetitions maximum (RM) in Stage 1 and 12 RM in Stage 2, with 15-second rest intervals between exercises and 2-minutes rest intervals between the two sets. The resistance exercise will comprise 10 exercises: reverse fly (pull apart), chest press, front raise, bicep curl, lateral trunk flexion, leg press, hip abduction, standing calf raise, squat, and lateral walk. The fitness coach will determine the initial resistance level of the bands according to each participant’s upper- and lower- limb functional capacity and will make monthly adjustments as needed. The procedures used to assess 15RM and 12RM in the present study will be adapted to Pan Z et al. [39]. Each elastic resistance band will be pre‑marked with 16 segments spaced at 10‑cm intervals. The midpoint of the band will be set as zero, with numbers 1–8 marked symmetrically on either side to form a standardized grip‑width scale. A narrower grip width will produce higher resistance. Participants will be instructed to adjust the grip width based on their strength capacity to determine the grip position corresponding to their 15RM or 12RM. The target grip width will be confirmed when participants reach volitional fatigue or muscular failure after completing 15 or 12 repetitions, respectively.

Before the intervention begins, the fitness coach will conduct a face-to-face training session to confirm that the participants have the ability to perform the exercises correctly and safely. A WeChat group will be established to facilitate communication and to provide intervention-related reminders and guidance. Five minutes before each exercise session, the fitness coach will send a reminder message to prompt participants to join the Tencent Meeting online room on time. During each session, the participants will be required to keep their web cameras on to enable real-time supervision and guidance. The heart rates will be continuously monitored using a Polar® Vantage V Titan (Polar Electro Oy, Kempele, Finland). After each session, the exercise intensity will be assessed using the Borg Scales for rating of perceived exertion (ranges from 6–20; 6 represents no exertion and 20 indicates maximal exertion) [40]. Attendance will be recorded at each session. If a participant experiences any adverse symptoms (e.g., injury, palpitations, hypoglycemia, pain, or dyspnea), the exercise session will be stopped immediately.

The HE group will receive a 30-minute health education course once per week for 12 weeks. Topics will include medication use, glucose monitoring, interpretation of key clinical indicators of T2DM, and recognizing signs of poor glycemic control.

### 2.5 Measurements

Demographic characteristics, medical history, medication use, anthropometric data (e.g., height, weight, waist circumference, and body composition), daily physical activity levels will be collected at baseline. The outcome measures will be assessed at pre- and post-intervention. Post-intervention assessments will be performed within 48–72 hours after the final exercise session. A standardized assessment procedure will be implemented in this study. Each measurement (cognitive assessments, MRI scanning, blood sampling and physical fitness tests) will be completed within its fixed assessment window in the morning. Cognitive function will be evaluated based on a battery of neuropsychological tests. Magnetic resonance imaging (MRI) scans will include T1‑weighted imaging, resting‑state functional MRI (rs‑fMRI), and diffusion tensor imaging (DTI). A consecutive 3-day food diary will be recorded before each assessment visit. An oral glucose tolerance test and physical fitness assessments will also be conducted. In addition, we will record the class, dose, and administration timing of all medications at each assessment time‑point.

#### 2.5.1 The primary outcome.

**Global Cognitive Function:** Global cognitive function will be measured using the MoCA, which is widely used internationally and has been validated in older adults [41,42]. MoCA scores range from 0 to 30, with higher scores indicating better global cognitive performance.

#### 2.5.2 The secondary outcomes.

**Cognitive Subdomains Assessments:** Cognitive subdomains will be assessed using a battery of neuropsychological tests, including N-back task, Color-Word Stroop Test, Attention Network Test (ANT), Trail Making Test (Parts A and B), and Digit Symbol Substitution Test.

Working memory will be assessed using the N-back task [43,44]. During the task, participants will view single digits (0–9, white font on a black background) presented in pseudorandom order. A total of nine blocks will be presented: three 0-Back, three 1-Back, and three 2-Back blocks. In the 0-Back condition, participants will be instructed to determine whether the digit displayed on the current screen is 9. In the 1-Back and 2-Back conditions, participants will be required to determine whether the current digit matches the digit presented one or two trials earlier, respectively. Each block will consist of 20 trials. Each stimulus will be presented for 1000ms, followed by a fixation cross (“+”) for 1000ms.

Inhibition function will be assessed using the Color-Word Stroop Test [45,46], in which participants will be required to identify the color of a word (red, green, yellow, or blue) and press the corresponding button as rapidly as possible. The participants will complete two blocks, totaling 192 trials. Each stimulus will be presented for 2000ms, followed by a fixation cross (“+”) for 1000ms.

The ANT was designed to assess three attention components: alerting, orienting, and executive control. The ANT protocol described by Fan et al. [47] will be adopted in this study. In ANT, a fixation cross (” + ”) will be displayed at the center of the screen. Each pilot consists of five arrows arranged horizontally or a single central arrow with two horizontal bars on each side. The central arrow (target) points left or right, and participants will be required to respond to the direction of the target arrow by pressing the keyboard “1” key to the left and “2” key to the right. The task included 288 trials, divided into three blocks. Adequate rest will be provided between the blocks. Participants will proceed to the next block after they report feeling sufficiently rested, thereby minimizing task‑related fatigue. As shown in Fig 3, at the beginning of each trial, a fixation cross (” + ”) will be presented for 400–1600 ms, followed by a 100-ms cue stimulus. The fixation will then reappear for 400ms. Next, the target stimulus will be presented, and participants must respond within 1700ms. A fixation cross (“+”) will appear immediately after the response, with a presentation duration depended on the reaction time (RT) to the target arrow and the initial fixation cross intertrial interval = 3500 ms – RT – duration of the first fixation cross. The ANT includes three target conditions (neutral, congruent, and incongruent) and four cue types (no cue, center cue, double cue, and spatial cue). Attention components are calculated as follows: 1) alerting = RT _No cue-_ RT _Double cue_, 2) orienting = RT _Center cue_ -RT _Spatial cue_, 3) executive control = RT _Incongruent_ -RT _Congruent._

**Fig 3:**
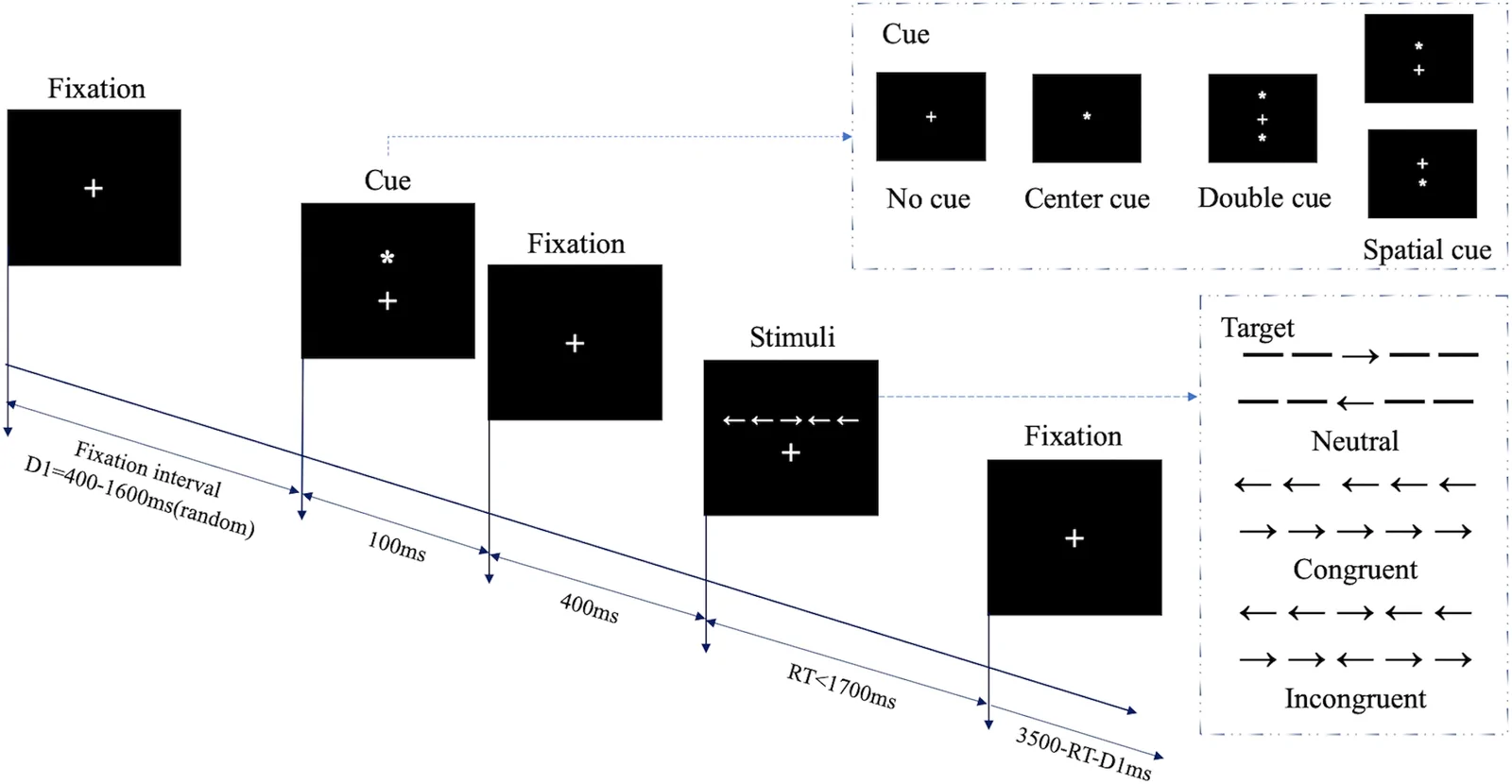
Attention Network Test.

Cognitive flexibility will be measured using the Trail Making Test (Part A and Part B) [48]. In Part A, the participants will be instructed to connect the numbers 1–25 sequentially on paper using a pen, completing the task as quickly as possible. For Part B, participants will be required to sequentially connect the numbers 1–25 (presented in alternating circles and squares) in ascending order as rapidly as possible, while alternating between the two shapes (----etc.) [42].

Processing speed will be assessed using the Digit Symbol Substitution Test [49]. The participants will be provided with a table containing numbers the 1–9 and their corresponding symbols. They will then be instructed to fill in the matching symbols next to the corresponding numbers in the table, completing as many correct entries as possible within 90s. Higher numbers of correctly filled symbols indicate better processing speed (i.e., higher scores).

**Magnetic Resonance Imaging:** All participants will undergo multimodal MRI scans at baseline and post-intervention using a 3.0 T Magnetom Prisma Scanner (Siemens, Erlangen, Germany) with a 64-channel head coil. In addition to the general informed consent, participants will sign a separate written informed consent form specifically for MRI scans. Table 2 presents the imaging sequences in acquisition order along with their detailed parameters, acquisition protocols, and corresponding outcomes. Participants will wear 3M 1110 high‑attenuation earplugs to reduce scanner‑induced noise. During the resting-state scan, participants will be instructed to remain awake, relaxed, and still; avoid thinking about anything in particular; and keep their eyes on a fixation cross (“+”) projected onto the screen.

**Table 2:** Imaging sequences, parameters, protocols, and outcomes.

Sequence	Main parameters	Outcomes	Scan duration
Resting-state EPI	TR = 2000ms, TE = 30ms; 62 slices, slice thickness = 2 mm, voxel size = 2.0 × 2.0 × 2.0 mm^3^, flip angle = 80°, FOV = 208 × 208 mm^2^	Brain activation and functional connectivity	8 min 21s
T1-weighted MPRAGE structural	TR = 2300ms, TE = 2.26 ms, 240 slices, time of inversion = 900ms, slice thickness = 1 mm, flip angle = 8°, voxel size = 1.0 × 1.0 × 1.0 mm^3^, FOV = 256 × 256 mm^2^	Grey matter volume, total intracranial volume, hippocampal volume and BrainAge	5 min 21s
Diffusion tensor imaging, EPI	TR = 5600ms, TE = 82ms, 99 slices, slice thickness = 1.5 mm, voxel size = 1.5 × 1.5 × 1.5 mm^3^, FOV = 210 × 210 mm^2^, 64 directions (*b* = 0/1000 s/mm^2^)	White matter microstructure	6 min 33s

Abbreviations: EPI, echo-echo planar imaging; MPRAGE, magnetization-prepared rapid gradient echo imaging; TR, repetition time; TE, echo time; FOV, field of view.

**MRI Data Processing:** As shown in Table 2, rs-fMRI data processing will be processed using MATLAB R2023b (MathWorks, Natick, MA, USA) and DPABI (http://rfmri.org/dpabi) [50]. The preprocessing steps will include:1) removing the first 10 images, 2) slice timing, realignment, 3) origin correction, 4) head motion correction, 5) spatial normalization to the Montreal Neurological Institute space using DARTEL, 6) regression of head motion parameters, white matter signal, cerebrospinal fluid signal, and global signal as covariates, 7) spatial smoothing using a Gaussian kernel with a full width at half maximum of 6 mm before the fractional amplitude of low-frequency fluctuation (fALFF) and after regional homogeneity (ReHo) calculation. ALFF and ReHo calculations for the low-frequency band (0.01–0.08 Hz), and transformation of both ALFF and ReHo values into a Z score (zALFF and zReHo) for further analysis [51,52]. Data with mean frame‑wise displacement (FD) > 0.3 mm will be excluded. Mean FD will be included as a covariate in the statistical analysis.

Seed-based whole-brain functional connectivity (FC) analysis: the bilateral hippocampus (defined from the Automated Anatomical Labeling 3 template) [53], the medial prefrontal cortex (MNI coordinates: [− 1,27,54], radius = 5 mm) and posterior cingulate cortex (MNI coordinates: [0, −52, 27], radius = 5 mm) will be used as a priori seed regions of interest [54]. The mean BOLD time course will be extracted from each seed, and Pearson’s correlation coefficients were calculated to correlate these time courses with whole-brain voxels. Finally, the FC maps will be normalized into z score maps by Fisher Z-transformation for subsequent analysis.

T1-weighted structural images will be preprocessed using the FreeSurfer Version 8.2.0 (https://surfer.nmr.mgh.harvard.edu/) longitudinal processing pipeline. Right and left hippocampal volumes will be extracted as a primary structural outcome. Other structural outcomes will be regard as exploratory analysis: 1) cortical thickness and surface area will be extracted based on the Desikan–Killiany atlas, while subcortical gray matter volumes will be derived using the ASEG atlas. BrainAge will be subsequently estimated via BrainAGE models of CentileBrain (https://centilebrain.org/#/); 2) voxel-based morphometry (VBM) analysis will be performed on the T1-weighted structural images using CAT12 implemented in SPM12 (The Wellcome Department of Cognitive Neurology, London, UK) to identify potential intervention-related differences in grey matter volume. CAT12’s longitudinal preprocessing pipelines, optimized for detecting small volumetric changes (i.e., the neuroplasticity workflow), will be adopted. A detailed description of the VBM data preprocessing can be found in the CAT12 Manual [55].

Diffusion tensor imaging (DTI) data will be analysed using FSL software (http://www.fmrib.ox.ac.uk/fsl/index.html). DTI data will be preprocessed, including brain extraction (“bet”), motion and eddy current correction (“eddy”) and tensor fitting (“dtifit”) to extracting values for fractional anisotropy (FA), mean diffusivity and radial diffusivity. Tract-based spatial statistical (TBSS) analysis will be employed to study WM changes at the voxel-to-voxel (whole-brain) level. All FA images will be registered to the FMRIB58_FA template. A skeleton map covering major common white‑matter tracts will be derived from the averaged FA image across all participants using the default threshold of 0.2. This skeleton map will subsequently be used to project FA data from each participant onto the mean skeleton. For TBSS, the number of permutations will be 5000 using threshold-free cluster enhancement. The statistical significance will be set to P  <  0.05.

**Anthropometric Assessment:** Bodyweight and height will be measured using a stadiometer (TANITA, Japan). Using a tape measure, waist circumference will be measured at the midpoint between the lower costal margin and the anterior superior iliac crests, and hip circumference will be measured at the level of the greater trochanter. Body composition will be measured by dual-energy X-ray absorptiometry (Lunar Prodigy GE Health, US). Blood pressure will be assessed twice using a digital sphygmomanometer (HEM-907-E, Omron, Tokyo, Japan).

**Oral Glucose Tolerance Test:** After the participants have fasted overnight, an intravenous catheter will be inserted into the antecubital vein by an experienced nurse for blood sampling. Blood samples will be analysed for glycated hemoglobin, glucose, insulin, C-peptide, triglycerides, total cholesterol, high-density lipoprotein cholesterol, low-density lipoprotein cholesterol, and free fatty acids. Participants will then consume a standard oral glucose tolerance test solution (75 g anhydrous glucose dissolved in 300 mL water) within 5 minutes. Additional blood samples will be collected at 30, 60, and 120 minutes post-ingestion. All samples will be left to stand at room temperature for 30 minutes and then centrifuged at 4°C and 3000 rpm for 15 minutes. The separated serum will be aliquoted into Eppendorf tubes and stored at −80°C until subsequent analysis.

**Physical Fitness Test:** Physical fitness will be assessed across five domains: muscle strength, muscle endurance, flexibility, balance, and cardiopulmonary endurance. Grip strength will be measured twice in each hand using a digital strain-gauge dynamometer (Takei TKK 5401; Takei Scientific Instruments, Tokyo, Japan). Lower-extremity strength will be evaluated using the 30s Chair-Stand Test [56]. The Timed Up and Go Test [57] will be used to assess walking ability, balance, and sit-to-stand transition capacity. Cardiopulmonary endurance will be evaluated using the 6-Minute Walk Test [58].

### 2.6 Data collection and management

This study will adhere to standardized testing procedures, with each outcome measure assessed by the same investigator to ensure consistency. A unique identification number will be assigned to each participant and used as the sole identifier throughout the data collection process. Paper-based documents, including baseline information forms, questionnaires, and assessment scales, will be securely stored in a locked filing cabinet within a restricted-access office. Two independent investigators will enter the final results into a password-protected computer and perform cross-verification; data access will be restricted to the principal investigator and lead researchers. As this study does not involve any pharmaceutical trials, sponsors and funding agencies will have no access to the raw data. Participants’ personal information will not be disclosed outside the research team without their written consent.

### 2.7 Adverse events

A pre-participation health screening will be conducted prior to enrollment to exclude those individuals who may be at high risk of exercise. This study uses a moderate-intensity exercise intervention, with certified fitness professionals providing continuous guidance and supervision throughout each session to ensure safety. To minimize the risk of hypoglycemia, all exercise sessions in the present study will be performed in the post‑prandial state. Participants in the EE group will receive additional safety education and training in self-monitoring. They will be counselled on the risk of nocturnal hypoglycemia and trained to recognize typical symptoms, including palpitations, night sweats, nocturnal awakening, and morning headaches. They will also be instructed to monitor their physical status following each exercise session. Should they experience any discomfort before bedtime, they should promptly measure their blood glucose and consume carbohydrates as needed. If hypoglycemia occurs following the first evening exercise session, the research team will evaluate the associated safety risk and discontinue further intervention if necessary. In addition, accident insurance will be purchased for all participants during the intervention period. However, as this study involves resistance exercises, participants may experience mild muscle soreness, which represents a transient physiological response to resistance exercises. All adverse events occurring during the exercise interventions will be assessed, categorized, and documented by the investigators to determine their causal relationship to the study. Reporting will follow the guidelines outlined in the Committees on Human Research “Adverse Event and Unanticipated Problems Reporting Policy”.

### 2.8 Statistical analysis

#### 2.8.1 Sample size calculation.

The sample size was estimated based on global cognitive outcomes reported in previous meta-analysis of multimodal exercise, which reported an effect size of approximately 0.5 for multimodal exercise [59,60]. A conservative effect size of Cohen’s f = 0.25 was adopted for the present study. Sample size estimation was performed using G*Power 3.1 for repeated-measures ANOVA (within–between interaction), with a two-tailed α of 0.05, statistical power of 0.80, a primary two-group comparison (ME and EE), and two assessment time points (pre- and post-intervention). The primary analysis required 34 participants. A matched control group was further enrolled to ensure design balance, resulting in 51 completers for three groups. Allowing for an attrition rate of 20%, a total of 66 participants will be enrolled in this study.

#### 2.8.2 Statistical analysis.

**Clinical and Behavioral Data Analysis:** Data will be analysed with SPSS 26.0. Two analytical approaches will be employed: per-protocol analysis and intention-to-treat analysis. Owing to the technical challenges and limited applicability of imputation methods in neuroimaging research, the neuroimaging dataset will be analysed using the per-protocol approach only. For the intention-to-treat principle, missing data will be handled using multiple imputation by chained equations (MICE), assuming that data are missing at random. We will impute 15 datasets, each with 10 iterations of the chained‑equations algorithm [61,62]. Imputations will be performed using the MICE package in R (version 4.5.0).

Descriptive statistics for continuous variables will be presented as mean (standard deviation) or median (interquartile range) for continuous variables. The Shapiro–Wilk test will be used to assess the normality of the continuous variables. One-way analysis of variance will be used to examine baseline differences among the three groups. Non-normally distributed data will be analysed using the Kruskal–Wallis rank-sum test. A linear mixed‑effects model (LMM) will be used to examine the effects of group (HE, ME, and EE), time (pre‑ and post‑intervention), and group × time interaction on cognition, metabolism and physical fitness outcomes. Group, time, and the group × time interaction will be regarded as fixed effects, and participant ID as a random intercept. Age, sex, diabetes duration, medication changes and education will be included as covariates. An unstructured covariance matrix will be used. Model parameters will be estimated using restricted maximum likelihood, and the Satterthwaite approximation will be applied for the degrees of freedom. For outcomes showing a significant group × time interaction, pre-specified simple-effects analyses based on estimated marginal means will be performed, with the resulting pairwise P-values Benjamini–Hochberg (BH)-corrected within the corresponding outcome domain (cognition, metabolism, or physical fitness). Within each domain, the Benjamini–Hochberg procedure will control the false discovery rate at 5%, and each domain will constitute an independent correction family. The 95% confidence intervals will be calculated for all outcomes and statistical significance will be set at a two-tailed P < 0.05. Moreover, according to minimal clinically important difference thresholds, an absolute reduction of ≥0.5% on the HbA1c, and an increase of ≥1 point on the MoCA score will be considered clinically meaningful.

**MRI Data Analysis:** The group analyses of fALFF, ReHo, FC, and VBM will be performed using the Flexible Factorial module in SPM12, with two factors: group (HE, ME, and EE) and time (pre- and post-intervention). The above-mentioned variables, including mean FD (for fALFF, ReHo, and FC analyses) and total intracranial volume (for VBM analysis), will be included as covariates. For whole-brain fMRI (fALFF, ReHo, and FC) and VBM, Gaussian random field theory correction threshold will be voxel level P  <  0.001 and cluster level P  <  0.05. Hippocampal volumes will be analysed using LMM. For TBSS, threshold-free cluster enhancement with 5,000 permutations will be applied, and statistical significance will be set at P < 0.05.

A further exploratory analysis will be to explore the associations between exercise-induced changes in brain structure or function and changes in cognitive function, partial correlation analysis will be performed. The Model 4 of the PROCESS macro for SPSS 26.0 will be used to examine the mediating effects of glycemic control (changes in HbA1c) on the relationship between exercise interventions and changes in cognitive function or brain health. Age, sex, diabetes duration, medication changes, and education will be included as covariates in the mediation models. Bias‑corrected bootstrapping with 5000 replications will be used to generate 95% confidence intervals for the direct and indirect effects. An effect will be considered statistically significant if the 95% CI did not include zero.

## 3. Discussion

This study aims to provide evidence-based and effective exercise recommendations for older adults with T2DM‐MCI, with the goal of maximizing the health benefits of exercise. The primary objective is to determine whether morning or evening exercise confers greater benefits to cognitive performance and brain health in this population. Furthermore, this study will examine whether changes in glucose mediate the effects of exercise on cognitive function, brain activation, and brain structure.

To date, research on the optimal exercise timing in T2DM‐MCI patients has focused predominantly on metabolism, and no consensus has been reached regarding the most beneficial time of day for exercise to improve glycemic control and lipid metabolism in this population. A recent meta-analysis showed that afternoon exercise is more effective than morning exercise in reducing fasting blood glucose levels and serum triglycerides in adults across varying health conditions [29]. Given the well-established association between glycemic status and cognitive performance, this study will not only provide evidence regarding the optimal exercise timing for cognitive benefits in T2DM‐MCI patients but will also explore whether the optimal exercise timing for metabolic benefits is consistent with that for cognitive benefits. Notably, previous studies have only compared cognitive improvements across different exercise times of day. To our knowledge, this study will be the first to expand on this research by investigating the differences in changes in brain function and structure using MRI.

This study protocol has limitations that could potentially impact the interpretation and applicability of the results. First, the absence of strict dietary control may confound the results, given the inherent challenges involved in modifying the long-established dietary patterns and calorie intake in older adults. Second, the imaging subsample will be non-random and likely underpowered, given that MRI-ineligible participants will remain in the study but will not undergo scanning. Third, requirements for smartphone proficiency and home internet access may select a relatively technology-literate subgroup of older adults, which may limit generalizability of findings. Fourth, the 3-month exercise intervention period may be insufficient to detect the full range of cognitive benefits. Whether longer exercise interventions (e.g., one year or more) would yield similar conclusions regarding the optimal time of day for exercise will remain uncertain. And as chronotype will not be assessed in this study, we cannot exclude the possibility that inter-individual differences in circadian phase contributed to the observed findings. Finally, our study will compare only morning and evening exercise and will not include an afternoon exercise session. Future studies should explore the effects of morning, afternoon, and evening exercise on cognitive function in T2DM‐MCI patients.

In conclusion, previous studies have indicated that both afternoon and evening exercise can lead to superior blood glucose control in adults across varying health conditions, and that glycemic control is closely associated with cognitive function. Building on this evidence, we propose the following hypotheses: (1) evening exercise will exert the most pronounced effect on improving cognitive function and brain health in T2DM‐MCI patients and (2) glycemic control will play a mediating role in the cognitive function improvements induced by exercise.

## Supporting information

S1 File SPIRIT checklist. (DOC)

S2 File Research protocol (Chinese). (DOCX)

S3 File Research Protocol (English). (DOCX)
