# Supplementary material for: Effects and mechanisms of exercise timing on cognitive function in older type 2 diabetes mellitus patients with mild cognitive impairment: A magnetic resonance imaging-based randomized controlled trial protocol
Source: PLoS One. 2026 Nov 4;21(11):e0361868. doi: 10.1371/journal.pone.0361868 (PMC13734470; doi:10.1371/journal.pone.0361868)
Supplement: Supplementary file 1 [file pone.0361868.s002.docx]

**研究方案**

1. **研究目的**

探究12周的早上vs晚上运动对2型糖尿病合并轻度认知功能障碍老年人认知功能的影响及其脑机制。

2. **研究对象**

参与者通过微信朋友圈、社区、受试者招募群、医院门诊等发布招募海报、口口相传进行招募。

**2.1 纳入标准**

（1）年龄处于 60-75 岁区间；

（2）临床确诊为 2 型糖尿病；

（3） 存在主观记忆障碍，蒙特利尔认知评估量表评分＜26 分，且简易精神状态检查表评分≥24 分；
（4） 生活完全能够自理，工具性日常生活活动能力量表评分≥15 分；
（5） 无 2 型糖尿病严重并发症；
（6） 过去 3 个月内处于身体活动不足状态（中高强度身体活动：每周中等强度活动≥150 分钟，或每周高强度活动≥75 分钟）。
    2.2 **排除标准**
（1） 患有神经退行性疾病（如痴呆、帕金森病、脑血管疾病等）；
（2） 存在其他可能影响干预效果与评估结果的躯体疾病，如严重心脏病、肝病、肾病等；
（3） 有反复低血糖病史；
（4） 患有精神疾病，汉密尔顿抑郁量表评分＞7 分；
（5） 有酒精依赖史；
（6） 存在运动功能障碍；

（7）教育年限不足 6 年。

**2.3 退出标准**

（1）参与者自愿退出；

（2） 研究者认为参与者不适合继续参加本研究；

（3）参与者在干预期间受伤。

**2.4 样本量**

预计纳入人数66人。

**3. 实验设计**

本研究为单盲随机对照试验，参与者完成所有基线测试后，使用计算机生成的随机数随机分配，分配比例为1:1:1。参与者至以下3组：1）早上运动组；2)晚上运动组；3)健康教育组。早上的运动时间在9:00-10:10，晚上的运动时间在19:00-20:10。运动组完成3次/周，持续12周运动干预。健康教育组每周一次30分钟的健康教育课程。在最后一次运动干预结束48h后进行干预后测试（干预后测试项目及流程同基线测试），以避免最后一次的运动对最终结果的持续影响。在整个研究期间，记录参与者的药物服用情况，要求参与者保持原有日常生活状态。

**4. 测试项目**

**4.1 基本情况和人体测量学**

调查基本情况，包括性别、年龄、身高、体重、腰围、臀围、病史、用药情况和认知水平（Montreal Cognitive Assessment和Mini-Mental State Examination）。三轴加速度计运动传感器Actigraph GT3X+监测实验前7天的身体活动情况并记录佩戴情况。双能X射线骨密度仪测量身体成份。电子血压计测血压。

**4.2口服糖耐量测试**

口服糖耐量测试是一种糖负荷试验。在清晨空腹采集空腹血液，口服溶于300ml水内的无水葡萄糖粉75g，在5min内饮完。从服用第一口糖水开始计时，在服糖后的0.5h、1h、2h采集静脉血测量2h 的血糖水平（使用留置针采集血液，每次采集血液约3~5ml）。在此期间，参与者不可进食、喝茶、和咖啡、不做剧烈运动，尽量静坐减少活动，但也无需绝对卧床。分析血糖、胰岛素、糖化血红蛋白、C肽、游离脂肪酸、血脂等血液指标。

**4.3认知功能测试**

基于电脑和软件E-prime进行认知功能测试，包括：Stroop, N-back, Attention Network Test。基于笔和纸进行Montreal Cognitive Assessment, Trail making Test和Digit Symbol Substitution Test。

**4.4功能性核磁共振扫描**

所有核磁共振操作均由上海体育学院心理学实验室具有相关资质人员进行操作。采用 SIEMENS MAGNETOM Prisma科研型 3T 超导磁共振获取大脑功能和脑结构数据。首先采集8min的静息态功能磁共振和约5min的T1加权脑结构扫描，随后进行约6min 的DTI扫描。

参与者体位：嘱参与者戴上 3M 1110 型高降噪耳塞以减少扫描中机器运行声响对参与者的影响，参与者平躺在磁共振仪器的床上，用头部固定托将参与者头部进行固定，让参与者感觉头部于舒适的体位，用海绵垫填塞头部固定托之间的空隙，降低机器震动对头部活动的影响同时能够尽量减少患者自主头部运动。

**4.5 健康体适能**

测试6分钟步行、握力、站立计时行走、30秒椅子站立。

**5. 运动干预方案**

本研究进行为期12周的中等强度有氧运动结合抗阻运动干预，每周进行3次（训练日不连续，每周一、三、五），每次运动约70min。有氧运动：40%-60% HRR可居家锻炼的无跳跃有氧健身操，动作包括原地踏步、踏步转手掌、前点屈肘、侧点手交叉踏步、侧点手拉弓踏步、平手抬膝等作为干预内容。全身抗阻运动：基于弹力带，以锻炼全身大肌肉群为主，针对胸背部肌群、肱二头肌、肱三头肌、腹部肌群、髋部肌群、臀部肌群、股二头肌和股四头肌等进行训练。共2组，一组10个动作，一个动作重复12-15次，每个动作休息15s，组别之间休息1min 。每个月定期增加适量弹力带磅数（依据受试者当前使用弹力带完成次数，使用当前弹力带完成动作超过 15 次则换弹性磅数更大的弹力带）。运动期间使用心率带监控运动强度。专门训练人员指导运动动作、监督运动干预过程并且记录出席次数。
