# Supplementary material for: Effects and mechanisms of exercise timing on cognitive function in older type 2 diabetes mellitus patients with mild cognitive impairment: A magnetic resonance imaging-based randomized controlled trial protocol
Source: PLoS One. 2026 Nov 4;21(11):e0361868. doi: 10.1371/journal.pone.0361868 (PMC13734470; doi:10.1371/journal.pone.0361868)
Supplement: Supplementary file 2 [file pone.0361868.s003.docx]

**Research Protocol**

**1. Research Purpose**

To investigate the effects of 12-week morning versus evening exercise on cognitive function and its underlying brain mechanisms in older adults with type 2 diabetes mellitus (T2DM) with mild cognitive impairment (MCI).

**2. Participants**

Participants will be recruited through multiple channels, including recruitment posters distributed via WeChat Moments, community centers, participant recruitment groups, and hospital outpatient clinics, as well as through word-of-mouth referrals.

**2.1 Inclusion Criteria**

(1) Aged 60–75 years;

(2) Clinically diagnosed with T2DM;

(3) Presence of subjective memory complaints, with a score < 26 on the Montreal Cognitive Assessment (MoCA) and a score ≥ 24 on the Mini-Mental State Examination (MMSE);

(4) Fully independent in daily living, with a score ≥ 15 on the Instrumental Activities of Daily Living (IADL) scale;

(5) No severe complications of T2DM;

(6) Insufficient physical activity in the past 3 months (defined as moderate-to-vigorous intensity physical activity ≥150 min/week of moderate intensity or ≥75 min/week of vigorous intensity physical activity).

**2.2 Exclusion Criteria**

(1) Diagnosis of neurodegenerative diseases (e.g., dementia, Parkinson’s disease, cerebrovascular diseases);

(2) Presence of other physical illnesses that may interfere with intervention effects or assessment results, such as severe heart disease, liver disease, or kidney disease;

(3) History of recurrent hypoglycemia;

(4) Diagnosis of mental illness, with a score > 7 on the Hamilton Depression Scale (HAMD);

(5) History of alcohol dependence;

(6) Presence of motor dysfunction;

(7) Educational attainment of < 6 years.

2.3 Withdrawal Criteria

(1) Voluntary withdrawal by the participant;

(2) Determination by the researcher that the participant is unsuitable to continue the study;

(3) Injury sustained by the participant during the intervention period.

**2.4 Sample Size**

A total of 66 participants are expected to be enrolled.

**3. Study Design**

This is a single-blind randomized controlled trial. After completing all baseline assessments, participants will be randomly assigned to one of three groups at a 1:1:1 ratio using computer-generated random numbers: (1) Morning Exercise Group, (2) Evening Exercise Group, and (3) Health Education Group. The Morning Exercise Group will conduct sessions between 9:00 and 9:10, while the Evening Exercise Group will exercise between 19:00 and 20:10. The health education group received a 30-minute health education session once a week. The exercise groups will receive 3 sessions per week for 12 consecutive weeks. Post-intervention assessments (with identical items and procedures to baseline assessments) will be conducted 48 hours after the final exercise session to avoid residual effects of the last exercise on study outcomes. Throughout the study, participants’ medication use will be documented, and they will be asked to maintain their usual daily living routines.

**4. Assessment Items**

**4.1 Basic Information and Anthropometry**

Basic information will be collected, including gender, age, height, weight, waist circumference, hip circumference, medical history, medication use, and cognitive function (assessed via the MoCA and MMSE). A triaxial accelerometer (Actigraph GT3X+) will be used to monitor participants’ physical activity levels in the 7 days prior to the study, with wearing status also recorded. Body composition will be measured using a dual-energy X-ray absorptiometry scanner, and blood pressure will be assessed with an electronic sphygmomanometer.

**4.2 Oral Glucose Tolerance Test (OGTT)**

The OGTT is a glucose load test conducted as follows: Fasting blood samples will be collected in the early morning. Participants will consume 75 g of anhydrous glucose powder dissolved in 300 mL of water within 5 minutes. Venous blood samples will be collected at 0.5, 1, and 2 hours after the first sip of the glucose solution to measure 2-hour blood glucose levels (an indwelling needle will be used for blood collection, with approximately 3–5 mL of blood drawn per sample).

During the test, participants will be instructed not to eat, drink tea or coffee, or engage in strenuous exercise. They should remain seated to minimize activity but are not required to stay in bed. Blood markers, including blood glucose, insulin, glycated hemoglobin (HbA1c), C-peptide, free fatty acids, and blood lipids, will be analyzed.

**4.3 Cognitive Function Assessments**

Computer-based tests (administered via E-Prime software): Stroop Test, N-back Test, and Attention Network Test. Paper-and-pencil tests: MoCA, Trail Making Test, and Digit Symbol Substitution Test.

**4.4 Functional Magnetic Resonance Imaging (fMRI) Scanning**

All MRI scans will be performed by qualified personnel from the Psychology Laboratory of Shanghai University of Sport using a SIEMENS MAGNETOM Prisma 3T research-grade superconducting MRI scanner. Scanning procedures will include: 8 minutes of resting-state fMRI, approximately 5 minutes of T1-weighted structural MRI and 6 minutes of diffusion tensor imaging.

Participant positioning: Participants will wear 3M 1110 high-noise-reduction earplugs to reduce scanner noise. They will lie supine on the MRI table, with their head secured using a head holder to ensure comfort. Sponge pads will be placed in the gaps of the head holder to reduce head movement caused by scanner vibration and minimize voluntary head motion.

**4.5 Physical Fitness Tests**

Tests will include the 6-Minute Walk Test, Grip Strength Test, Timed Up and Go Test, and 30-Second Chair Stand Test.

**5. Exercise Intervention**

The 12-week intervention will combine moderate-intensity aerobic exercise and resistance training, with 3 non-consecutive sessions per week and each session lasting approximately 70 minutes. Aerobic exercise will consist of jump-free aerobic routines suitable for home-based practice, performed at 40%–60% heart rate reserve. Exercises will include: in-place stepping, stepping with palm rotation, forward stepping with elbow flexion, side stepping with hand crossing, side stepping with bow-and-arrow arm movement, and knee lifting with flat hands. Whole-body resistance training will use elastic bands and target major muscle groups, including the chest, back, biceps brachii, triceps brachii, abdomen, hips, glutes, biceps femoris, and quadriceps femoris. The training protocol will include: 2 sets of 10 exercises each, 12–15 repetitions per exercise, 15 seconds of rest between exercises, 1 minute of rest between sets. The tension of the elastic bands will be increased monthly based on participants’ performance: if a participant can complete more than 15 repetitions of an exercise with the current band, a band with higher tension will be used. Heart rate monitors will be used to monitor exercise intensity during sessions. Professional trainers will guide participants on exercise form, supervise the intervention process, and record attendance.
